# COVID-19 in the years 2020 to 2022 in Germany: effects of comorbidities and co-medications based on a large-scale database analysis

**DOI:** 10.1186/s12889-024-21110-7

**Published:** 2025-02-08

**Authors:** Roland Linder, Jonas Peltner, Anatoli Astvatsatourov, Willy Gomm, Britta Haenisch

**Affiliations:** 1 Techniker Krankenkasse, Hamburg, Germany; 2 German Center for Neurodegenerative Diseases (DZNE) e.V, Bonn, Germany; 3 Clinical Trials Division, Federal Institute for Drugs and Medical Devices, Bonn, Germany; 4 Research Division, Federal Institute for Drugs and Medical Devices, Kurt-Georg-Kiesinger-Allee 3, 53175 Bonn, Germany; 5 Center for Translational Medicine, Medical Faculty, University of Bonn, Bonn, Germany

**Keywords:** Case-control study, COVID-19, Coronavirus, SARS-CoV-2, Epidemiology, Claims data

## Abstract

**Background:**

The SARS-CoV-2 pandemic was a challenge for health care systems worldwide. People with pre-existing chronic diseases have been identified as vulnerable patient groups. Furthermore, some of the drugs used for these chronic diseases such as antihypertensive drugs have been discussed as possible influencing factors on the progression of COVID-19. This study examines the effect of medication- and morbidity-associated risk factors suspected to moderate the disease course and progression of COVID-19.

**Methods:**

The study is based on claims data of the Techniker Krankenkasse, Germany’s largest statutory health insurance. The data cover the years 2020 to 2022 and include insured persons with COVID-19 diagnosis from both the outpatient and inpatient sectors and a control of insured persons without COVID-19 diagnosis. We conducted a matched case-control study and matched each patient with an inpatient diagnosis of COVID-19 to (a) 10 control patients and (b) one patient with an outpatient diagnosis of COVID-19 to form two study cohorts. We performed a descriptive analysis to describe the proportion of patients in the two cohorts who were diagnosed with comorbidities or medication use known to influence the risk of COVID-19 progression. Multiple logistic regression models were used to identify risk factors for disease progression.

**Results:**

In the first study period the first study cohort comprised a total of 150,018 patients (13,638 cases hospitalised with COVID-19 and 136,380 control patients without a COVID-19 infection). Study cohort 2 included 27,238 patients (13,619 patients hospitalised with COVID-19 and 13,619 control patients with an outpatient COVID-19 diagnosis). Immunodeficiencies and use of immunosuppressives were strongest risk modifying factors for hospitalization in both study populations. Other comorbidities associated with hospitalization were diabetes, hypertension, and depression.

**Conclusion:**

We have shown that hospitalisation with COVID-19 is associated with past medical history and medication use. Furthermore, we have demonstrated the ability of claims data as a timely available data source to identify risk factors for COVID-19 severity based on large numbers of patients. Given our results, claims data have the potential to be useful as part of a surveillance protocol allowing early-stage access to epidemiological data in future pandemics.

**Supplementary Information:**

The online version contains supplementary material available at 10.1186/s12889-024-21110-7.

## Background

The SARS-CoV-2 virus first emerged in China at the end of 2019 and escalated into a global pandemic in the following year [1, 2]. The disease caused by the virus, coronavirus disease 2019 (COVID-19), has a wide range of severity and symptoms [3]. The disease can be accompanied by pneumonia and acute respiratory distress syndrome, so that artificial ventilation may be necessary [4, 5].

Systematic reviews and cohort studies from many countries have identified several comorbidities such as diabetes and cardiovascular disease as risk factors for COVID-19 disease severity [6–11]. For example, three studies from Germany [12–14] which used electronic hospital records and claims data, identified hypertension, cardiac disease, diabetes, and chronic obstructive pulmonary disease (COPD) as factors associated with severe infections or COVID-19 related mortality. However, these studies use, as most of the studies conducted in other countries as well, only hospitalised cases and did not include infected patients who were not hospitalised or a control group of uninfected patients [15]. Including only hospitalised cases might bias study results as hospitalised cases represent more severe COVID-19 progressions and might not be representative of the disease burden in the general population. Furthermore, most studies only covered a relatively short time span during the year 2020 although later studies suggested that characteristics of patients hospitalised due to COVID-19 changed during the course of the pandemic [16, 17].

In this study we describe the characteristics of patients in Germany hospitalised with COVID-19 compared to matched controls without a COVID-19 infection and matched controls with an outpatient diagnosis of COVID-19 in the period from January 2020 to December 2022 in a German population of patients insured by statutory health insurance. Comorbidities and medications associated with increased risk for severe COVID-19 infections or death are investigated. Furthermore, we aim to identify risk factors for hospitalisation with COVID-19 by comparing patients hospitalised with COVID-19 to healthy controls and patients with mild COVID-19 infections.

## Methods

### Data source

We performed a non-interventional, retrospective case-control study using claims data from the *Techniker Krankenkasse* (TK), a German statutory health insurance company, which insured about 11.6 million patients at the time of writing in July 2024. The legal basis for the cooperation between the different institutions involved in the analyses is a cooperation agreement in accordance with Sect. 75 of the German Social Security Code V (transmission of social data for research and planning). The planned data transfer from the TK to the external organisations was submitted to the Federal Office for Social Security (BAS) for review and approved. To prevent re-identification, exact dates of diagnoses were excluded from the data and time-related information as only available in relation to the study entry date. Certain information such as a participant’s zip code was also coarsened to further prevent re-identification. The BAS weighed the expected benefits of the study against the data protection risks and determined that the remaining residual risk of re-identification and data misuse is acceptable.

The data extraction period spanned January 2019 to June 2023. The data included patients’ date of birth, self-reported gender, weekly inpatient diagnoses and quarterly outpatient diagnoses, hospitalisations, care levels and ambulatory drug reimbursement. Ambulatory drug reimbursement data were extracted between January 2019 and June 2022, and included the date of dispensing and the ATC codes. Medical in- and outpatient diagnoses were coded using the German version of ICD-10. COVID-19 diagnoses were used for the time period from January 2020 to December 2022. Data related to comorbidities were used from January 2019 to June 2022.

We defined six study periods for the analyses by dividing the period beginning January 2020 and ending in December 2022 into half-year periods (H1/2020, H2/2020, H1/2021, H2/2021, H1/2022, and H2/2022). We chose six study periods covering six months each in order to better capture the infection process, the epidemiological events, the wave-like course of the pandemic, and the beginning and course of the vaccination campaign.

### Study cohorts

We included three groups of patients for each of the six study periods in our analyses: Patients with at least one hospitalisation with COVID-19 in the study period (COVID-19 With Hospitalisation, C19WH). Main and secondary diagnoses with ICD-10-GM primary and secondary codes U07.1! and U07.2! were used to define hospitalisation with COVID-19. U07.2! was used in addition to U07.1! to also capture infections which have been confirmed clinically or epidemiologically. For the C19WH group the exact day of the hospital admission was available. The second group included patients with an outpatient diagnosis of COVID-19 (ICD-10-GM primary and secondary codes U07.1! and U07.2!; COVID-19 WithOut Hospitalisation, C19WOH). For this group only the quarter of the outpatient diagnosis was available. The third group comprised control patients without a COVID-19 infection during the respective study period (CONTRL).

We defined the cohort entry date (CED) in each study period as the first date of the first hospital admission with COVID-19 (C19WH) or as the quarter with the first outpatient COVID-19 diagnosis (C19WOH). The year preceding the quarter of the CED was used for assessing comorbidities and medication use for all patients. It was possible for a patient to be included in multiple cohorts, if they met the inclusion criteria at multiple assessment points. As only the first hospital admission or outpatient diagnosis in each study period was allowed as the CED, a patient who was admitted to a hospital after a re-infection or received a second outpatient diagnosis due to a re-infection in a study period, was not able to enter the cohort twice in that period. This approach also prevented an erroneous duplication of patients among those who were hospitalized with COVID-19 and whose infection was also recorded in the outpatient sector. Patients who were already hospitalised with COVID-19 at the beginning of the study period were eligible for inclusion as cases only in the period in which the infection occurred.

Patients from the C19WH and C19WOH groups were excluded from the analysis if they were not consecutively insured with the TK for the period starting in the year before the CED (i.e. the covariate assessment window) until 90 days after the CED (i.e. the follow-up window used to assess mortality, which was used in analyses not included in the present study). Controls had to be continuously insured with the TK for the same period as their match to be included in the study. Furthermore, patients who objected the use of their data for research purposes were excluded.

We performed a retrospective matched case-control study and defined two study cohorts for our analyses: In all study periods each patient from the C19WH was matched to 10 CONTRL patients using cumulative density sampling (study cohort 1). The exact matching was performed by the TK before data delivery. Each case in the C19WH group was assigned 10 controls of the same age and sex without replacement. Age (year of birth for the matching procedure) was assigned to the exact year for all cases with appropriate controls in 1:10 matching. Additionally, controls were required to be alive for a certain period of time after the CED of their matched case. Other characteristics were not included in the matching and it was assumed that those were randomly and evenly distributed between cases and controls. To form the second study cohort, each C19WH patient was matched to one C19WOH patient using, again, age and sex as matching variables. For this cohort a 1:1 matching approach was used as there were not enough C19WOH patients in the data to perform a 1:10 matching.

### Exposure variables

Exposure variables included several comorbidities and co-medications for which previous studies found evidence supporting their modifying the occurrence, course of the disease and progression of COVID-19 infections [18–22]. For a comorbidity to be counted as present, at least one in- or outpatient diagnosis had to be recorded for a patient in the year preceding the CED. All ICD-10-GM codes used to define each comorbidity are listed in tables S1 and S2 in the supplementary material. Medication use was assessed using redeemed prescriptions. To be counted as a user of a certain medicine, a patient had to have at least one redeemed prescription for that medicine in the year before the CED. Age, sex, and level of care were used as potential confounders in the statistical analyses. Grade of care was defined as a categorical variable with five levels according to the German long-term care insurance act [23]. Higher grades of care indicate more severe impairment. A detailed description of the five levels can be found in table S3 in the supplementary material.

### Statistical analysis

A descriptive analysis was performed to estimate the distribution of comorbidities, co-medications and the clinical outcome by study cohort in the first study period. Categorical variables were expressed by percentages. Conditional multivariate logistic regression analysis was used to estimate the association between hospitalization with COVID-19 and the exposure variables, and level of care was included as an additional confounding in both study cohorts. Matching groups were used as stratification variables. Results for HIV are not presented due to the small number of cases (i.e. less than 0.4% of the study population).

**Fig. 1:**
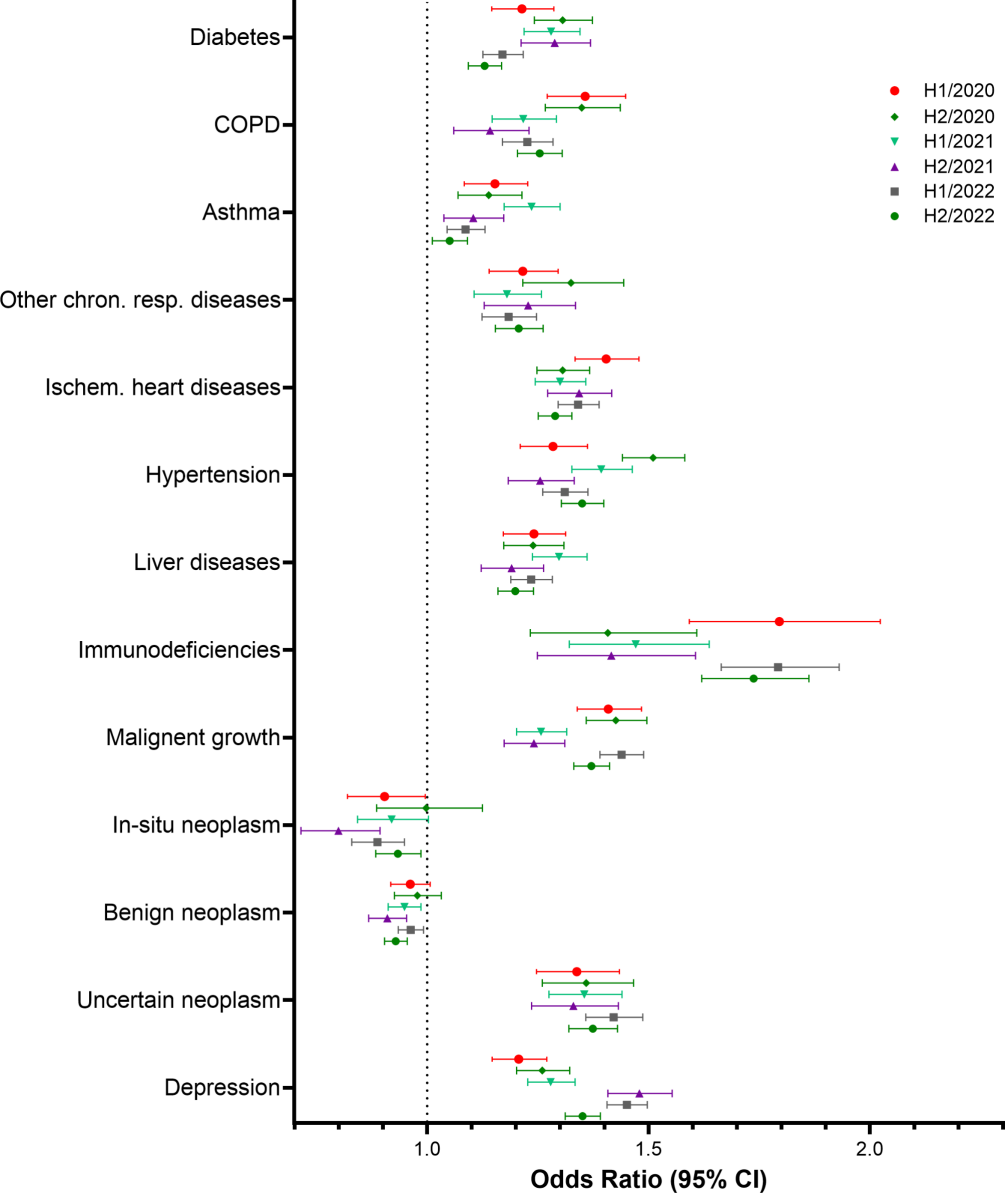
Results of the multivariate logistic regression. Study cohort 1. Odds Ratios and 95% confidence limits for comorbidities

**Fig. 2:**
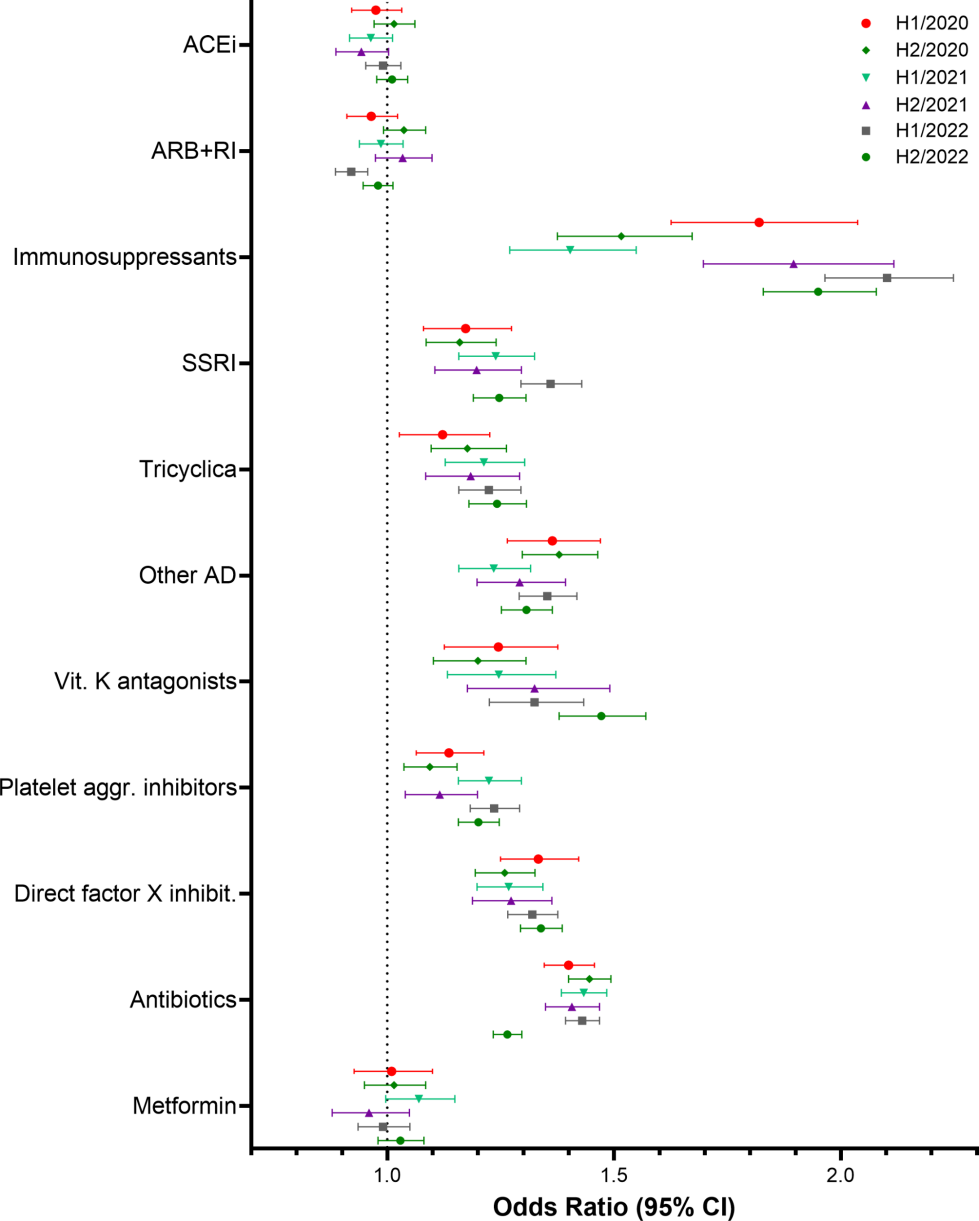
Results of the multivariate logistic regression. Study cohort 1. Odds Ratios and 95% confidence limits for comedications

**Fig. 3:**
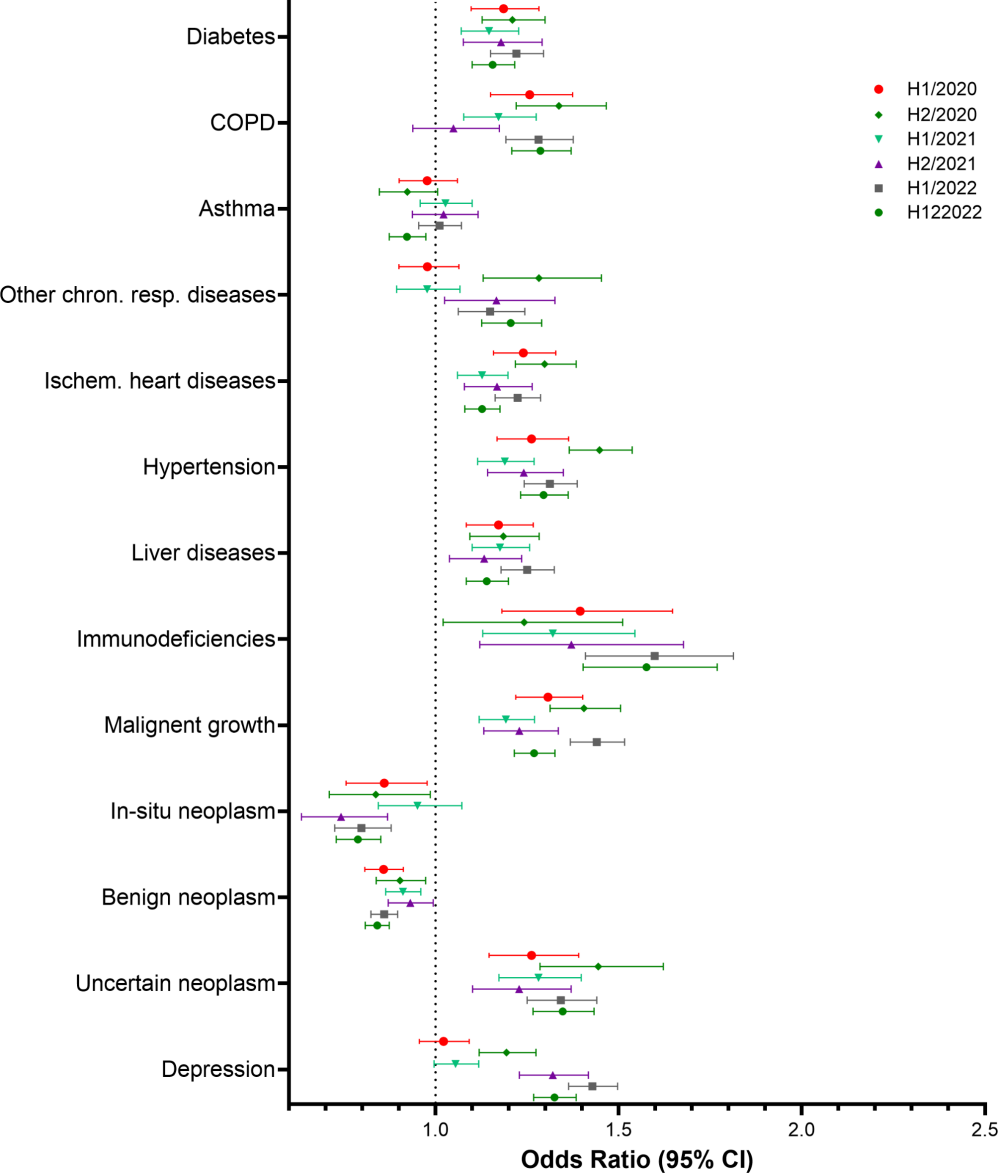
Results of the multivariate logistic regression. Study cohort 2. Odds Ratios and 95% confidence limits for comorbidities

**Fig. 4:**
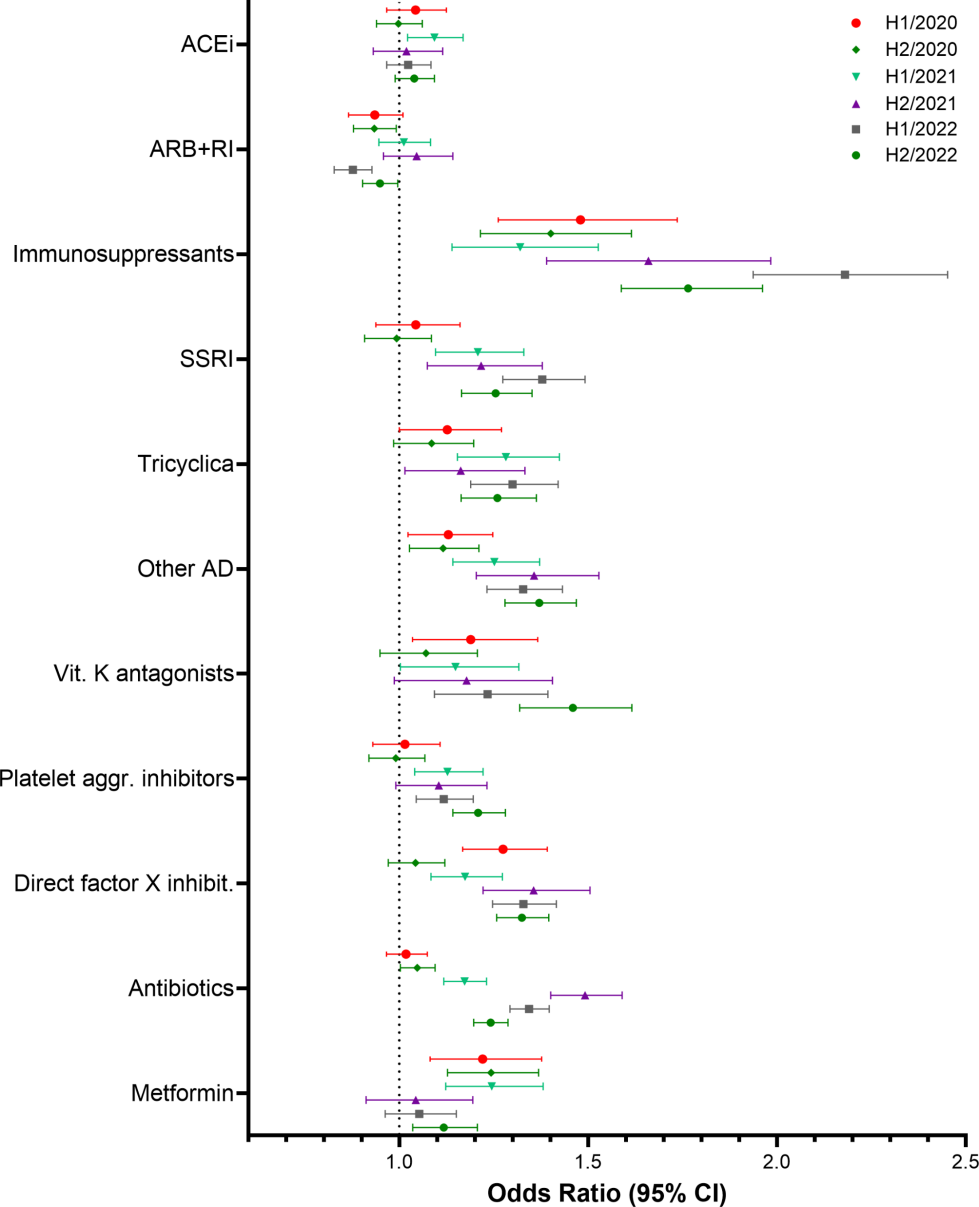
Results of the multivariate logistic regression. Study cohort 2. Odds Ratios and 95% confidence limits for comedications

**Fig. 5:**
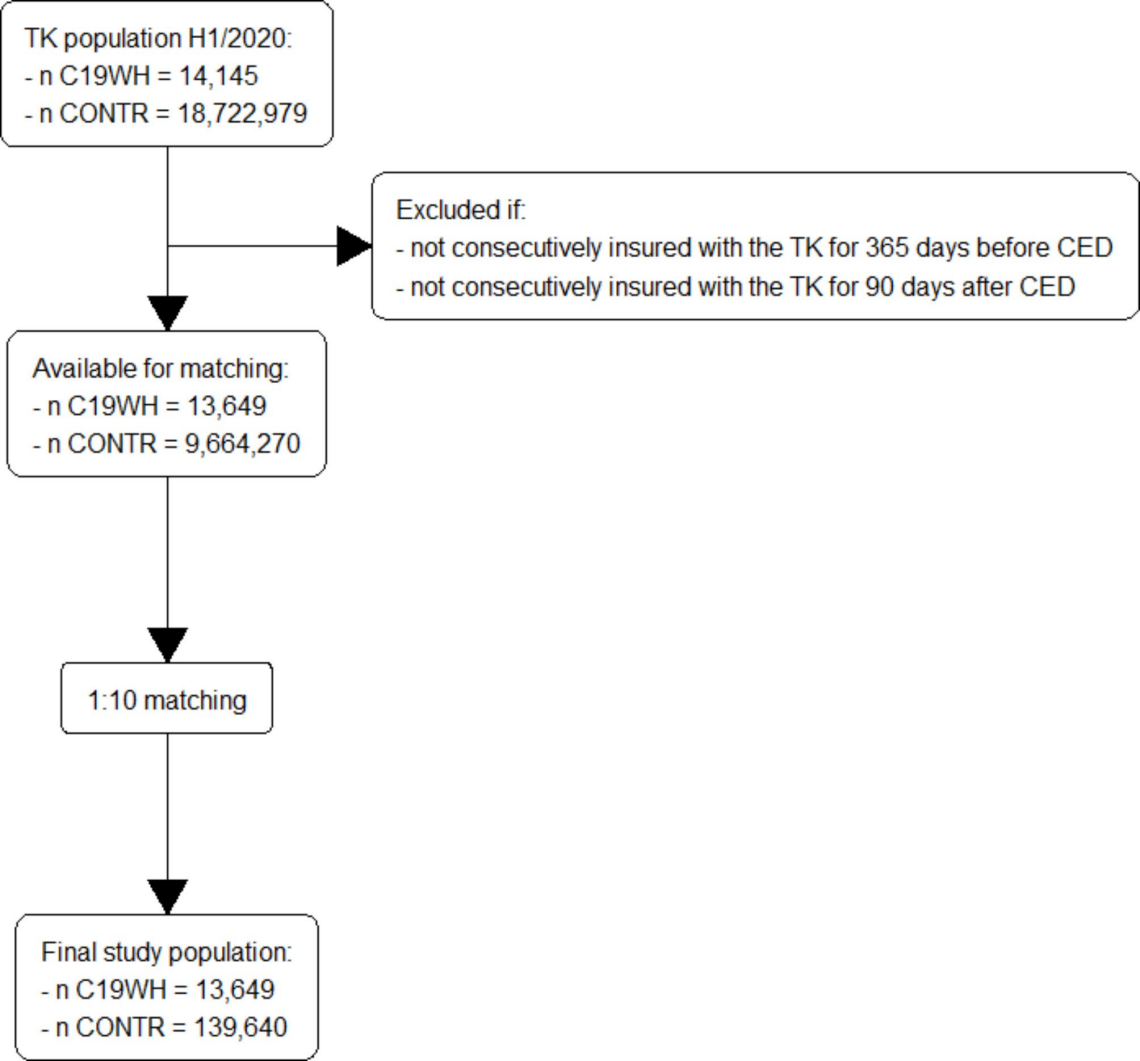
Study flow, study period 1

Descriptive and multivariate analyses were performed using SAS software (SAS Institute Inc., Cary, NC, USA), version 9.4. Figures 1, 2, 3, and 4 were created using GraphPad Prism version 10.0.0 for Windows, GraphPad Software, Boston, Massachusetts USA. Figure 5 was created using R version 4.4.1 (R Foundation for Statistical Computing, Vienna, Austria).

## Results

In the first study period 14,145 C19WH patients and 18,722,979 CONTROL patients were available to form study cohort 1 (Fig. 5). Application of the inclusion and exclusion criteria reduced the number of patients available for the matching process to 13,649 C19WH and 9,664,270 CONTROL patients. After the matching process, the final study population for study cohort 1 comprised 13,649 C19WH and 136,490 CONTROL patients. For study cohort 2, 14,145 C19WH patients and 200,750 C19WOH patients were available before inclusion and exclusion criteria were applied and 13,649 C19WH and 185,751 C19WOH patients were included in the matching. The final study cohort included 13,627 C19WH patients and 13,627 matched C19WOH patients. Cohort attrition for all other study periods is described in supplementary table S4.

A total of 13,649 C19WH (44% female), 13,627 C19WOH (45% female), and 136,490 CONTRL (45% female) patients were included in the first study period, which covered the first half of 2020. As shown in Table 1, more males than females (7,578 vs. 6,091) were hospitalised with COVID-19 or had an outpatient COVID-19 diagnosis (7,558 vs. 6,069). The age distribution shows that male cases were older compared to female cases and that most cases came from the group aged 60 to 79 years. In females, a relatively high proportion of younger patients aged 15 to 34 years had a hospital admission with COVID-19 in the first study period.

**Table 1:** Age distribution of cases and controls stratified by sex, first study period (H1/2020)

Agegroup	Female			Male		
	C19WH	C19WOH	CONTRL	C19WH	C19WOH	CONTRL
0–14	198 (3.26%)	198 (3.26%)	1980 (3.26%)	224 (2.96%)	224 (2.96%)	2240 (2.96%)
15–34	838 (13.80%)	838 (13.81%)	8380 (13.80%)	579 (7.64%)	579 (7.66%)	5790 (7.64%)
35–59	1691 (27.85%)	1691 (27.86%)	16,910 (27.85%)	1871 (24.69%)	1870 (24.74%)	18,710 (24.69%)
60–79	1763 (29.04%)	1763 (29.05%)	17,630 (29.04%)	2767 (36.51%)	2767 (36.61%)	27,670 (36.51%)
80+	1581 (26.04%)	1579 (26.02%)	15,810 (26.04%)	2137 (28.20%)	2118 (28.02%)	21,370 (28.20%)

Note: C19WH: patients with an inpatient diagnosis of COVID-19 during the study period. C19WOH: patients with an outpatient diagnosis of COVID-19 during the study period. CONTRL: patients without a COVID-19 diagnosis during the study period

Hypertension was the most common comorbidity in females in all three groups with 52.0% of C19WH, 44.7% of C19WOH, and 43.5% of female CONTRL patients being diagnosed with hypertension in the baseline period (Table 2). Other common comorbidities included depression, ischemic heart disease, and benign neoplasms. With the exception of benign neoplasms, all comorbidities were more common in C19WH females compared to the matched C19WOH females. Notable differences in the proportions of patients with a comorbidity were observed for diabetes (C19WH: 18.1%, C19WOH: 12.0%) and immunodeficiencies (C19WH: 3.1%, C19WOH: 1.8%). Similarly, all comorbidities, with the exception of in-situ neoplasms, were more common in C19WH females than in the matched CONTRL females. COPD (C19WH: 11.5%, CONTRL: 5.5%) and immuodeficiencies (C19WH: 3.1%, CONTRL: 1.2%) were notably more common in C19WH females. Use of all studied medications was more common in C19WH females than in the two other groups. ACE inhibitors, ARB + RI, and antibiotics were the most prescribed drugs in all three groups of females.

**Table 2:** Comparison of hospitalised cases, patients with outpatient COVID-19 diagnosis and uninfected controls by sex

	Female			Male		
	C19WH (*n* = 6071)	C19WOH (*n* = 6069)	CONTRL (*n* = 60710)	C19WH (*n* = 7578)	C19WOH (*n* = 7558)	CONTRL (*n* = 75780)
*Comorbidities*						
Diabetes	1101 (18.1%)	766 (12.6%)	7301 (12.0%)	2037 (26.9%)	1536 (20.3%)	13,895 (18.3%)
COPD	701 (11.5%)	475 (7.8%)	3339 (5.5%)	1070 (14.1%)	711 (9.4%)	5160 (6.8%)
Asthma	776 (12.8%)	704 (11.6%)	5524 (9.1%)	777 (10.3%)	724 (9.6%)	5473 (7.2%)
Other chronic respiratory diseases	677 (11.2%)	607 (10.0%)	4058 (6.7%)	908 (12.0%)	799 (10.6%)	5091 (6.7%)
Ischemic heart disease	1656 (27.3%)	1253 (20.6%)	10,543 (17.4%)	2978 (39.3%)	2387 (31.6%)	20,806 (27.5%)
Hypertension	3158 (52.0%)	2711 (44.7%)	26,381 (43.5%)	4747 (62.6%)	4241 (56.1%)	40,436 (53.4%)
Liver diseases	731 (12.0%)	517 (8.5%)	4862 (8.0%)	1207 (15.9%)	959 (12.7%)	8301 (11.0%)
HIV	3 (0.0%)	2 (0.0%)	23 (0.0%)	28 (0.4%)	31 (0.4%)	120 (0.2%)
Immunodeficiencies	188 (3.1%)	108 (1.8%)	723 (1.2%)	257 (3.4%)	133 (1.8%)	783 (1.0%)
Malignant growth	1118 (18.4%)	811 (13.4%)	7568 (12.5%)	1915 (25.3%)	1580 (20.9%)	13,701 (18.1%)
In-situ neoplasm	172 (2.8%)	158 (2.6%)	1803 (3.0%)	396 (5.2%)	461 (6.1%)	4008 (5.3%)
Benign neoplasm	1363 (22.5%)	1446 (23.8%)	13,444 (22.1%)	1615 (21.3%)	1785 (23.6%)	16,037 (21.2%)
Neoplasm of uncertain behavior	498 (8.2%)	342 (5.6%)	2936 (4.8%)	761 (10.0%)	567 (7.5%)	4468 (5.9%)
Depression	2018 (33.2%)	1729 (28.5%)	12,971 (21.4%)	1651 (21.8%)	1485 (19.6%)	9748 (12.9%)
*Comedications*						
ACEi	1100 (18.1%)	907 (14.9%)	9218 (15.2%)	2002 (26.4%)	1723 (22.8%)	17,204 (22.7%)
ARB + RI	1179 (19.4%)	1066 (17.6%)	10,459 (17.2%)	1702 (22.5%)	1636 (21.6%)	15,374 (20.3%)
Immunosuppressants	216 (3.6%)	100 (1.6%)	735 (1.2%)	320 (4.2%)	191 (2.5%)	1035 (1.4%)
SSRI	541 (8.9%)	475 (7.8%)	2673 (4.4%)	446 (5.9%)	365 (4.8%)	1956 (2.6%)
Tricyclic antidepressants	452 (7.4%)	346 (5.7%)	2850 (4.7%)	281 (3.7%)	233 (3.1%)	1724 (2.3%)
Other antidepressants	669 (11.0%)	520 (8.6%)	3065 (5.0%)	604 (8.0%)	452 (6.0%)	2412 (3.2%)
Vitamin K antagonists	165 (2.7%)	125 (2.1%)	1060 (1.7%)	399 (5.3%)	301 (4.0%)	2902 (3.8%)
Platelet aggregation inhibitors	521 (8.6%)	398 (6.6%)	2993 (4.9%)	1168 (15.4%)	972 (12.9%)	7336 (9.7%)
Direct factor Xa inhibitors	678 (11.2%)	472 (7.8%)	3511 (5.8%)	1078 (14.2%)	773 (10.2%)	6342 (8.4%)
Antibiotics	2469 (40.7%)	2267 (37.4%)	17,223 (28.4%)	2805 (37.0%)	2536 (33.6%)	17,797 (23.5%)
Metformin	326 (5.4%)	200 (3.3%)	2197 (3.6%)	711 (9.4%)	483 (6.4%)	5086 (6.7%)

Notes: C19WH: patients with an inpatient diagnosis of COVID-19 during the study period. C19WOH: patients with an outpatient diagnosis of COVID-19 during the study period. CONTRL: patients without a COVID-19 diagnosis during the study period. COPD: chronic obstructive pulmonary disease. HIV: human immunodeficiency virus. ACEi: angiotensin-converting enzyme inhibitor. ARB + RI: angiotensin II receptor blockers and renin inhibitors. SSRI: selective serotonin reuptake inhibitor. ICD codes and ATC codes used to define comorbidities and medications can be found in supplementary tables S1 and S2

In male patients, the most common comorbidities included hypertension (C19WH: 4,747 (62.6%), C19WOH: 4,241 (56.1%), CONTRL: 40,436 (53.4%)), ischemic heart disease (C19WH: 2,978 (39.3%), C19WOH: 2,387 (31.6%), CONTRL: 20,806 (27.5%)), and diabetes (C19WH: 2,037 (26.9%), C19WOH: 1,536 (20.3%), CONTRL: 13,895 (18.3%)). Excepting in-situ neoplasms, benign neoplasms, and HIV, comorbidities were more common in C19WH patients when compared to C19WH and C19WOH patients. Similarly, compared to CONTRL patients, C19WH patients were more often diagnosed with all comorbidities except in-situ neoplasms. Among all medications assessed in male patients, antibiotics (C19WH: 2,805 (37.0%), C19WOH: 2,536 (33.6%), CONTRL: 17,797 (23.5%)), ACE inhibitors (C19WH: 2,002 (26.4%), C19WOH: 1,723 (22.8%), CONTRL: 17,204 (22.7%)), and ARB + RI (C19WH: 1,702 (22.5%), C19WOH: 1,636 (21.6%), CONTRL: 15,374 (20.3%)) were most commonly used in all groups. Notably, C19WH patients used more immunosuppressants than the other two groups.

### Risk factors for hospitalisation with COVID-19 – study cohort 1

Figures 1 and 2 show the results of the multivariate stratified logistic regression for the study cohort of C19WH patients and their matched controls for each study period and a table presenting the data used to create the figures can be found in the supplementary material, tables S5 and S6. In Fig. 1, odds ratios and their corresponding 95%-CIs for the included comorbidities are reported and Fig. 2 shows odds ratios and 95%-CIs for the investigated medications. All assessed comorbidities, with the exceptions of in-situ neoplasms and benign neoplasms, were associated with an increased risk of hospitalisation with COVID-19 and odds ratios mostly ranged from 1.15 (95% CI [1.08;1.23]) for asthma to 1.80 (95% CI [1.59;2.02]) for immunodeficiencies. Comparing the six study periods, revealed no overarching time trend. Patients hospitalised with COVID-19 had higher odds of use of most medications under study compared to the uninfected controls. For use of ACE inhibitors, ARB + RI, and metformin the results were inconclusive as the 95%-CIs indicate that odds ratios smaller than one and odds ratios larger than one are compatible with the data. Not all odds ratios for medications correspond well to the odds ratios of the diseases for which they are indicated. For example, odds ratios for depression ranged from about 1.21 (95% CI [1.15;1.27]) in study period 1 to 1.48 (95% CI [1.41;1.53]) in study period 4 and odds ratios for SSRIs, tricyclic antidepressants and other antidepressants used to treat depression are of about the same magnitude. On the other hand, hypertension and diabetes were found to increase the risk of hospitalisation, while for medications to treat these conditions, i.e. metformin, ACE inhibitors, and ARB + RI, the results were inconclusive.

A patient’s impairment was also associated with the risk of hospitalisation with COVID-19 in all study periods (figure S1). Odds ratios for patients with care grade 5 which implies the most severe level of impairment of independence or capabilities compared to patients with no impairment ranged from 6.58 (95% CI [5.60;7.72]) in study period 4 to 8.80 (95% CI [7.60;10.09]) in study period 1.

### Risk factors for hospitalisation with COVID-19 – study cohort 2

Results of the conditional logistic regression performed in the second study cohort which included only patients with a COVID-19 infection are displayed in Figs. 3 and 4. In this cohort, most comorbidities were associated with increased odds of experiencing the outcome in all study periods. In-situ neoplasms and benign neoplasms were associated with a lower risk of hospitalisation. For asthma no clear association could be established. Similar to the first study cohort, there was no apparent temporal pattern. A temporal trend was only indicated for depression, as the odds ratios in the last three study periods are notably higher compared to the first three study periods. Most medications were associated with higher odds of experiencing the outcome in most study periods. However, the effects were less pronounced than in study cohort 1. Use of antidepressants was associated with higher odds of hospitalisation with COVID-19 in almost all study periods.

Effects of the grade of care were less pronounced in study cohort 2 compared to study cohort 1. In all study periods, patients with some level of impairment had higher risk of hospitalisation with COVID-19 than patients without impairment (figure S2). With the exception of care grade 1 there was also a visible time trend. Odds ratios increased in the first three study periods and remained relatively stable in the last three study periods.

## Discussion

To our knowledge, this study is the first to examine the clinical characteristics, medication use, and risk factors associated with hospitalisation with COVID-19 using a large national sample of patients over a long period of time covering most of the pandemic years in Germany. We included comorbidities and medications to treat those that were less commonly investigated in previous analyses to provide further evidence based on a large number of cases and controls for those groups.

In the first study period which covered the first half of 2020, comorbidities, including hypertension, ischemic heart disease, diabetes, and depression, were common among all groups of patients with some – such as diabetes – being more common in male patients than in females and others – such as depression – being more common in females than in males. These findings are similar to previous studies from both Germany [12–14] and other countries [7–10, 15, 24, 25] which also identified hypertension and diabetes as common comorbidities in patients with a COVID-19 infection. We also identified depression as a comorbidity which over proportionally affected female patients. Furthermore, our study revealed that immunodeficiencies were a comorbidity that disproportionately affected C19WH patients.

The comparability of the results of our multivariate analyses to those of previous studies is limited by differences in study design and outcomes. While most previous studies use only patients hospitalized with COVID-19 and use outcome measures such as death or ventilation, our study includes population controls (study cohort 1) or controls with mild infections (study cohort 2) and uses hospitalization with COVID-19 as the outcome of interest. Despite these differences, the results of the multivariate analyses confirm that risk factors identified by previous studies from Germany [12–14], Europe, Canada and the US [7, 9, 15, 24–28], or by meta-analyses [6, 8, 11] are associated with increased risk of COVID-19 severity. Odds ratios for commonly identified risk factors such as diabetes (range study cohort 1: 1.13 to 1.29; range study cohort 2: 1.14 to 1.22) or hypertension (range study cohort 1: 1.25 to 1.51; range study cohort 2: 1.19 to 1.44) were similar to those of a German study which also includes the general population, uses claims data andsimilar outcomes [13] and studies from Sweden and the UK who used a similar study design including population controls [15, 26].

Depression and use of antidepressants were both independently associated with an increased risk of hospitalization with COVID-19 in most study periods and in both study cohorts. Depression has been identified as a risk factor for COVID-19 severity in a prospective cohort study and a recent review [29], which identified an association between mood disorders (including depression and bipolar disorders) and COVID-19 severity as well as COVID-19 hospitalization. Findings for all classes of antidepressant drugs were in line with a study from Scotland using a similar design [30]. However, they contradict the result of studies that focused specifically on use of antidepressants and the COVID-19 course. This study found that SSRIs, especially fluvoxamine, was associated with lower disease severity [31–34]. A possible explanation for this difference could be that the association found in our study is confounded by using drug classes instead of patient drugs, which can differ widely in their pharmacological profile.

Another group of drugs that is being discussed as a possible modifying factor in the course of a COVID-19 infection and which has not been studied widely in claims data, are immunosuppressants. On the one hand, in some SARS-CoV-2 patients a situation develops that is compatible with a secondary virus-triggered hemophagocytic lymphohistiocytosis, whereby an excessive immune response to the infection could be the decisive factor [20]. On the other hand, immunosuppressive therapy can inhibit an antiviral immune reaction to respiratory viruses and thus intensify the viral disease [21]. Both points of view have a reasonable number of studies supporting them as shown in a recent systematic review including 22 studies [35], while an earlier meta-analysis including six studies with relatively small numbers of cases reported that immunosuppressed patients were not at an increased risk of COVID-19 infection [36]. Immunodeficiency and use of immunosuppressants were associated with the highest odds of hospitalisation with COVID-19 in our analyses although the estimates showed a larger variability than the estimates for other comorbidities and medications due to low numbers of affected patients. Our findings contrast those of previous studies with smaller sample sizes, but are in line with recently published - and thus more in line with the current assessment of this drug class-cohort studies with larger sample sizes [37, 38], which report that certain immunosuppressants such as glucocorticoids [38] or Janus kinase inhibitors [37] were associated with increased risk of severe illness or death. However, effect of patient drug classes assessed in these studies was inconsistent. These findings highlight that not all immunosuppressants equally influence the course of COVID-19 disease. We used an aggregated group of immunosuppressants and although we were able to confirm the results of the cohort studies, the possibility remains that some classes of immunosuppressants do not affect the course of COVID-19 disease but might have a protective effect.

### Strengths and limitations

Our study has several strengths. Using the TK claims data, we were able to draw cases and controls for our analyses from a large sample of the German population and include mild cases, severe cases and patients without infection for our analyses. The data also covered a two-year time span which allowed us to assess risk factors at different time points intervals during the pandemic. Since claims data are used for billing purposes, our data are of high quality and there is a low likelihood of misclassified or missing diagnoses, although diagnosis codes can be missing if, especially in a hospital setting, there is no impact on remuneration.

As each encounter with the healthcare system is documented in our data, we could assess comorbidities and used medications and include both in our statistical models. This allowed us to assess the effects of comorbidities independently from the effects of the medications used to treat them (and vice versa). Altogether, these strengths highlight the potential of claims data to serve as an additional resource for a fast identification of risk factors during newly emerging epidemics or pandemics. Many results of studies with small sample sizes could not be verified when using large amounts of data. Therefore, it is all the more important to establish suitable monitoring with a view to the next pandemic in order to be able to access large numbers at an early stage. Our results demonstrate the potential that claims data have as a data source for such a monitoring.

There are also several limitations to our data source and study design that have to be considered when interpreting the results. One limitation is the data source which only includes patients insured by the TK, Germany’s largest statutory health insurance. Furthermore, the regional uneven distribution of insured persons across the country, which leads to a small number of cases in some regions and insufficient representation, also prevented the carrying out of regionally differentiated analyses. However, our data include over 11 million insured persons, resulting in a large sample of the German population. We used hospital admission date as the CED to identify C19WH patients. This makes our study prone to outcome misclassification bias as patients who were already hospitalized with COVID-19 at the beginning of a study period could not be identified as cases but were misclassified as controls. Similarly, C19WOH patients with an outpatient diagnosis in the last quarter of a study period who were still infected in the following study period could also not be identified as cases in our study and were misclassified. Furthermore, due to using secondary diagnosis codes to identify patients hospitalised with COVID-19, it is possible that we included patients without severe symptoms as cases which could cause our estimates to be biased downwards. However, by also including secondary diagnoses, our inclusion criteria became more robust against diagnosis-related groups (DRG) gaming, which refers to inadequate revenue optimization within the DRG set of rules [39]. Thus, using this approach was more likely to include all patients with an infection. In inpatients with a secondary diagnosis code of COVID-19, the associated main diagnosis was mostly related to involvement of the respiratory tract or an (viral) infection, so that in these patients it can also be assumed that the reason for admission was related to COVID-19. Throughout the study, we used the term “hospitalized with COVID-19” in the manuscript to highlight that the hospital admission was COVID-19-related but not necessarily caused by COVID-19.

Information on drug dispensing was used as a proxy of exposure. Thus, non-adherence may be present in some cases, which leads to exposure misclassification and can result in bias towards the null. Information on lifestyle factors and health-related behavior such as obesity and smoking status tend to be greatly underreported in claims data which makes the corresponding ICD-codes unsuitable for the present analysis. Obesity and smoking have been identified as important risk factors for poor COVID-19 outcomes [6, 40, 41] and thus we cannot rule out residual confounding in our analyses. A further source of residual or unmeasured confounding is the retrospective design we chose for our study. Furthermore, claims data lack information on laboratory results and vital signs which could have been used to adjust for disease severity or to include more information in our model than simple indicators of presence or absence of a disease. Lastly, the generalisability of our findings might be limited due to a healthcare system in Germany that was not overburdened by the COVID-19 pandemic to a degree similar to the healthcare systems in other countries and different measures taken to tackle the pandemic in different countries.

## Conclusion

This study confirms that risk of hospitalisation with COVID-19 is associated with comorbidities such as diabetes, hypertension, immunodeficiency, and others as well as use of medications such as antidepressants, and immunosuppressants. These associations remained relatively stable over the course of the pandemic and when comparing patients hospitalised with COVID-19 to uninfected patients or patients with outpatient COVID-19 diagnoses. We were able to identify these risk factors and confirm previous German and international studies using claims data. Assuming timely access, this data can serve as a good source of information on millions of people to identify vulnerable populations in the event of a new pandemic or health crisis.

## Electronic supplementary material

Below is the link to the electronic supplementary material.

Supplementary Material 1

## Data Availability

The data that support the findings of this study are available from the Techniker Krankenkasse but restrictions apply to the availability of these data, which were used under license for the current study, and so are not publicly available.

## Abbreviations

ACEi: Angiotensin-converting enzyme inhibitor
ARB + RI: Angiotensin II receptor blocker and renin inhibitors
SSRI: Selective serotonin reuptake inhibitor
ATC: Anatomical therapeutic chemical classification
CED: Cohort entry date
CONTRL: Patients without a COVID-19 diagnosis during the study period
COPD: Chronic obstructive pulmonary disease
COVID-19: Coronavirus disease 2019
C19WH: Patients with inpatient diagnosis of COVID-19 during the study period
C19WOH: Patients with an outpatient diagnosis of COVID-19 during the study period
HIV: Human immunodeficiency virus
ICD-10: International statistical classification of diseases and related health problems, 10th revision
ICD-10 -GM: International statistical classification of diseases and related health problems, 10th revision, German modification
OR: Odds ratio
SD: Standard deviation
TK: Techniker Krankenkasse
