# Supplementary material for: COVID-19 in the years 2020 to 2022 in Germany: effects of comorbidities and co-medications based on a large-scale database analysis
Source: BMC Public Health. 2025 Feb 8;25:525. doi: 10.1186/s12889-024-21110-7 (PMC11806888; doi:10.1186/s12889-024-21110-7)
Supplement: Supplementary file 1 — Supplementary Material 1 [file 12889_2024_21110_MOESM1_ESM.docx]

**COVID-19 in the years 2020 to 2022 in Germany: effects of comorbidities and co-medications based on a large-scale database analysis**

**Supplementary material**

Roland Linder^1^, Jonas Peltner^2^, Anatoli Astvatsatourov^3^, Willy Gomm^2^, Britta Haenisch^4,2,5^

^1^Techniker Krankenkasse, Hamburg, Germany

^2^German Center for Neurodegenerative Diseases (DZNE) e.V., Bonn

^3^Clinical Trials Division, Federal Institute for Drugs and Medical Devices, Bonn, Germany

^4^Research Division, Federal Institute for Drugs and Medical Devices, Bonn, Germany

^5^Center for Translational Medicine, Medical Faculty, University of Bonn, Bonn, Germany

Table S1: Definitions of comorbidities used in the descriptive and multivariate analyses.

| **Comorbidity** | **ICD-10 GM** |
| --- | --- |
| Human immunodeficiency virus (HIV) disease | B20-B24 |
| Malign neoplasms | C00-C97 |
| In situ neoplasms | D00-D09 |
| Benign neoplasms | D10-D36 |
| Neoplasms of unspecified behavior | D37-D48 |
| Disorders involving the immune mechanism | D80-D90 |
| Diabetes mellitus | E10-E14 |
| Hypertension | I10-I13, I15 |
| Ischemic heart disease | I20-I25, I49-I50 |
| Chronic obstructive pulmonary disease | J44 |
| Asthma | J45 |
| Other chronic lower respiratory diseases | J40-J43, J46-J47 |
| Depression | F32-F34, F38-F39 |
| Liver diseases | K70-K77 |

Table S2: Definitions of medications used in the descriptive and multivarite analyses.

| **Medikamentengruppe** | **ATC** |
| --- | --- |
| Metformin | A10BA02 |
| Vitamin K antagonists | B01AA |
| Platelet aggregation inhibitors | B01AC |
| Direct factor X inhibitors | B01AF |
| ACE-inhibitors | C09A, C09B |
| Angiotensin II receptor blockers and renin inhibitors (ARB+RI) | C09C, C09D, C09X |
| Antibiotics | J01 |
| Immunosuppressants | L04, J05AR |
| Tricyclic antidepressants | N06AA |
| Selective serotonin reuptake inhibotors | N06AB |
| Other antidepressants | N06A excl. N06AA and N06AB |

Table S3: Grades of care according to the German long-term insurance act.

| **Grade of care** | **Description** |
| --- | --- |
| 1 | Low level of impairment of independence or capabilities |
| 2 | Significant level of impairment of independence or capabilities |
| 3 | Serious level of impairment of independence or capabilities |
| 4 | The most severe level of impairment of independence or capabilities |
| 5 | The most severe level of impairment of independence or capabilities with special long-term care requirements |

Table S4: Study flow and cohort attrition.

|  | Study period | | | | | |
| --- | --- | --- | --- | --- | --- | --- |
|  | 1 | 2 | 3 | 4 | 5 | 6 |
| *Study cohort 1* |  |  |  |  |  |  |
| Individuals with hospitalisation withCOVID-19^1^ | 14 145 | 22 061 | 20 359 | 14 875 | 37 915 | 40 579 |
| Available control individuals^1 2^ | 18 722 979 | 17 777 820 | 18 507 178 | 18 116 703 | 16 865 337 | 17 646 075 |
| *Matching process* |  |  |  |  |  |  |
| Number of cases used in the matching process | 13 649 | 20 929 | 19 404 | 13 881 | 34 947 | 38 137 |
| Number of controls used in the matching process | 9 664 270 | 9 279 584 | 9 634 077 | 9 548 225 | 8 264 266 | 9 054 040 |
| ***Final study population*** |  |  |  |  |  |  |
| Number of cases | 13 649 | 20 929 | 19 418 | 13 881 | 34 947 | 38 137 |
| Number of controls | 136 490 | 209 290 | 194 180 | 138 810 | 349 468 | 379 760 |
| *Study cohort 2* |  |  |  |  |  |  |
| Individuals with hospitalisation withCOVID-19^1^ | 14 145 | 22 061 | 20 359 | 14 875 | 37 915 | 40 579 |
| Available control individuals^1 3^ | 200 750 | 759 155 | 496 912 | 648 313 | 2 054 506 | 1 313 129 |
| *Matching process* |  |  |  |  |  |  |
| Number of cases used in the matching process | 13 649 | 20 929 | 19 404 | 13 881 | 34 947 | 38 137 |
| Number of controls used in the matching process | 185 751 | 703 124 | 455 625 | 603 951 | 1 934 122 | 1 244 688 |
| ***Final study population*** |  |  |  |  |  |  |
| Number of cases | 13 627 | 20 924 | 19 417 | 13 881 | 34 946 | 38 136 |
| Number of controls | 13 627 | 20 924 | 19 417 | 13 881 | 34 946 | 38 136 |

Notes: Study period 1: H1/2020, study period 2: H2/2020, study period 3: H1/2021, study period 4: H2/2021, study period 5: H1/2022, study period 6: H2/2022.^1^Before applying inclusion and exclusion criteria (see study cohorts section in the manuscript).^2^Individuals without a COVID-19 infection in the study period. ^3^Individuals with an outpatient diagnosis of COVID-19 in the study period.

TableS5a: Results of the multivariate logistic regression. Study cohort 1. Study periods 1 and 2.

|  | Study period 1 (n = 150 139) | | Study period 2 (n = 230 219) | |
| --- | --- | --- | --- | --- |
|  | OR | 95%CI | OR | 95%CI |
| *Comorbidities* |  |  |  |  |
| Diabetes | 1.214 | [1.146;1.286] | 1.306 | [1.242;1.373] |
| COPD | 1.357 | [1.271;1.448] | 1.349 | [1.267;1.436] |
| Asthma | 1.153 | [1.084;1.227] | 1.139 | [1.070;1.214] |
| Other chronic respiratory diseases | 1.216 | [1.140;1.296] | 1.325 | [1.216;1.444] |
| Ischemic heart disease | 1.404 | [1.334;1.478] | 1.306 | [1.248;1.367] |
| Hypertension | 1.284 | [1.210;1.362] | 1.510 | [1.441;1.582] |
| Liver diseases | 1.241 | [1.172;1.313] | 1.239 | [1.173;1.309] |
| Immunodeficiencies | 1.795 | [1.592;2.023] | 1.408 | [1.233;1.609] |
| Malignant growth | 1.409 | [1.339;1.484] | 1.426 | [1.359;1.496] |
| In-situ neoplasm | 0.904 | [0.820;0.996] | 0.998 | [0.886;1.125] |
| Benign neoplasm | 0.962 | [0.918;1.007] | 0.978 | [0.926;1.032] |
| Neoplasm of uncertain behavior | 1.338 | [1.247;1.434] | 1.359 | [1.260;1.466] |
| Depression | 1.207 | [1.147;1.270] | 1.260 | [1.202;1.322] |
| *Comedications* |  |  |  |  |
| ACE | 0.975 | [0.922;1.032] | 1.015 | [0.971;1.061] |
| ARB+RI | 0.965 | [0.911;1.023] | 1.037 | [0.992;1.085] |
| Immunosuppressants | 1.820 | [1.626;2.037] | 1.516 | [1.375;1.672] |
| SSRI | 1.173 | [1.080;1.274] | 1.160 | [1.086;1.240] |
| Tricyclic antidepressants | 1.122 | [1.027;1.226] | 1.177 | [1.097;1.263] |
| Other antidepressants | 1.364 | [1.265;1.470] | 1.379 | [1.298;1.464] |
| Vitamin K antagonists | 1.245 | [1.126;1.376] | 1.200 | [1.102;1.306] |
| Platelet aggregation inhibitors | 1.136 | [1.064;1.213] | 1.094 | [1.037;1.154] |
| Direct factor Xa inhibitors | 1.333 | [1.250;1.422] | 1.259 | [1.194;1.326] |
| Antibiotics | 1.400 | [1.346;1.457] | 1.446 | [1.400;1.493] |
| Metformin | 1.010 | [0.927;1.100] | 1.015 | [0.950;1.085] |
| *Grade of care* |  |  |  |  |
| Care grade 1 | 2.385 | [2.078;2.738] | 2.185 | [1.959;2.439] |
| Care grade 2 | 2.543 | [2.341;2.761] | 3.008 | [2.821;3.207] |
| Care grade 3 | 4.885 | [4.495;5.308] | 4.526 | [4.236;4.836] |
| Care grade 4 | 6.495 | [5.862;7.197] | 7.020 | [6.485;7.599] |
| Care grade 5 | 8.798 | [7.675;10.085] | 8.302 | [7.414;9.298] |

Notes: Study period 1: H1/2020. Study period 2: H2/2020.

Table S5b: Results of the multivariate logistic regression. Study cohort 1. Study periods 3 and 4.

|  | Study period 3 (n = 213 598) | | Study period 4 (n = 152 691) | |
| --- | --- | --- | --- | --- |
|  | OR | 95%CI | OR | 95%CI |
| *Comorbidities* |  |  |  |  |
| Diabetes | 1.280 | [1.219;1.345] | 1.288 | [1.212;1.369] |
| COPD | 1.217 | [1.147;1.292] | 1.142 | [1.060;1.230] |
| Asthma | 1.236 | [1.174;1.300] | 1.104 | [1.038;1.173] |
| Other chronic respiratory diseases | 1.180 | [1.106;1.258] | 1.228 | [1.129;1.335] |
| Ischemic heart disease | 1.300 | [1.244;1.358] | 1.343 | [1.272;1.417] |
| Hypertension | 1.393 | [1.327;1.463] | 1.255 | [1.183;1.332] |
| Liver diseases | 1.298 | [1.238;1.361] | 1.191 | [1.122;1.263] |
| Immunodeficiencies | 1.471 | [1.321;1.637] | 1.416 | [1.249;1.606] |
| Malignant growth | 1.257 | [1.202;1.315] | 1.241 | [1.174;1.311] |
| In-situ neoplasm | 0.920 | [0.843;1.003] | 0.800 | [0.715;0.894] |
| Benign neoplasm | 0.949 | [0.912;0.986] | 0.910 | [0.868;0.954] |
| Neoplasm of uncertain behavior | 1.355 | [1.275;1.440] | 1.330 | [1.236;1.432] |
| Depression | 1.279 | [1.227;1.334] | 1.479 | [1.408;1.553] |
| *Comedications* |  |  |  |  |
| ACE | 0.964 | [0.917;1.012] | 0.943 | [0.887;1.003] |
| ARB+RI | 0.986 | [0.939;1.035] | 1.034 | [0.974;1.099] |
| Immunosuppressants | 1.403 | [1.270;1.549] | 1.896 | [1.697;2.117] |
| SSRI | 1.239 | [1.158;1.325] | 1.197 | [1.105;1.296] |
| Tricyclic antidepressants | 1.213 | [1.128;1.303] | 1.184 | [1.085;1.292] |
| Other antidepressants | 1.235 | [1.158;1.316] | 1.292 | [1.198;1.393] |
| Vitamin K antagonists | 1.246 | [1.133;1.372] | 1.325 | [1.177;1.491] |
| Platelet aggregation inhibitors | 1.224 | [1.157;1.296] | 1.116 | [1.040;1.199] |
| Direct factor Xa inhibitors | 1.268 | [1.198;1.343] | 1.273 | [1.188;1.363] |
| Antibiotics | 1.433 | [1.384;1.484] | 1.407 | [1.349;1.468] |
| Metformin | 1.070 | [0.997;1.149] | 0.960 | [0.879;1.049] |
| *Grade of care* |  |  |  |  |
| Care grade 1 | 2.017 | [1.786;2.278] | 1.835 | [1.596;2.111] |
| Care grade 2 | 2.736 | [2.550;2.936] | 2.817 | [2.593;3.061] |
| Care grade 3 | 4.155 | [3.863;4.468] | 3.939 | [3.606;4.303] |
| Care grade 4 | 6.568 | [6.000;7.191] | 5.909 | [5.288;6.602] |
| Care grade 5 | 6.620 | [5.800;7.556] | 6.579 | [5.604;7.723] |

Note: Study period 3: H1/2021, study period 4: H2/2021.

Table S5c: Results of the multivariate logistic regression. Study cohort 1. Study periods 5 and 6.

|  | Study period 5 (n = 384 415) | | Study period 6 (n = 417 736) | |
| --- | --- | --- | --- | --- |
|  | OR | 95%CI | OR | 95%CI |
| *Comorbidities* |  |  |  |  |
| Diabetes | 1.170 | [1.126;1.217] | 1.130 | [1.093;1.168] |
| COPD | 1.226 | [1.170;1.284] | 1.254 | [1.204;1.305] |
| Asthma | 1.087 | [1.045;1.131] | 1.051 | [1.012;1.091] |
| Other chronic respiratory diseases | 1.184 | [1.124;1.247] | 1.207 | [1.154;1.262] |
| Ischemic heart disease | 1.341 | [1.296;1.388] | 1.289 | [1.251;1.327] |
| Hypertension | 1.311 | [1.261;1.363] | 1.350 | [1.303;1.399] |
| Liver diseases | 1.235 | [1.189;1.283] | 1.199 | [1.160;1.240] |
| Immunodeficiencies | 1.792 | [1.664;1.930] | 1.737 | [1.620;1.862] |
| Malignant growth | 1.439 | [1.390;1.489] | 1.371 | [1.331;1.412] |
| In-situ neoplasm | 0.888 | [0.830;0.949] | 0.934 | [0.884;0.986] |
| Benign neoplasm | 0.963 | [0.935;0.992] | 0.929 | [0.904;0.955] |
| Neoplasm of uncertain behavior | 1.421 | [1.358;1.487] | 1.374 | [1.320;1.430] |
| Depression | 1.451 | [1.406;1.497] | 1.351 | [1.312;1.391] |
| *Comedications* |  |  |  |  |
| ACE | 0.991 | [0.953;1.030] | 1.011 | [0.977;1.045] |
| ARB+RI | 0.921 | [0.886;0.957] | 0.980 | [0.947;1.013] |
| Immunosuppressants | 2.102 | [1.965;2.248] | 1.950 | [1.829;2.078] |
| SSRI | 1.360 | [1.295;1.429] | 1.247 | [1.190;1.306] |
| Tricyclic antidepressants | 1.224 | [1.158;1.295] | 1.242 | [1.180;1.307] |
| Other antidepressants | 1.353 | [1.291;1.418] | 1.307 | [1.252;1.364] |
| Vitamin K antagonists | 1.325 | [1.225;1.433] | 1.472 | [1.379;1.570] |
| Platelet aggregation inhibitors | 1.236 | [1.183;1.292] | 1.201 | [1.157;1.247] |
| Direct factor Xa inhibitors | 1.320 | [1.266;1.376] | 1.339 | [1.294;1.386] |
| Antibiotics | 1.430 | [1.393;1.468] | 1.265 | [1.234;1.297] |
| Metformin | 0.991 | [0.936;1.050] | 1.029 | [0.980;1.081] |
| *Grade of care* |  |  |  |  |
| Care grade 1 | 2.056 | [1.894;2.233] | 2.130 | [1.991;2.278] |
| Care grade 2 | 3.100 | [2.948;3.259] | 3.046 | [2.921;3.177] |
| Care grade 3 | 4.973 | [4.723;5.237] | 4.700 | [4.499;4.910] |
| Care grade 4 | 7.451 | [6.975;7.960] | 6.369 | [6.019;6.740] |
| Care grade 5 | 8.367 | [7.617;9.190] | 7.091 | [6.517;7.716] |

Note: Study period 5: H1/2022, study period 6: H2/2022.

Table S6a: Results of the multivariate logistic regression. Study cohort 2. Study periods 1 and 2.

|  | Study period 1 (n = 27 254) | | Study period 2 (n = 41 848) | |
| --- | --- | --- | --- | --- |
|  | OR | 95%CI | OR | 95%CI |
| *Comorbidities* |  |  |  |  |
| Diabetes | 1.186 | [1.097;1.282] | 1.210 | [1.127;1.299] |
| COPD | 1.257 | [1.150;1.374] | 1.337 | [1.220;1.466] |
| Asthma | 0.977 | [0.901;1.060] | 0.923 | [0.847;1.006] |
| Other chronic respiratory diseases | 0.978 | [0.900;1.064] | 1.282 | [1.130;1.453] |
| Ischemic heart disease | 1.240 | [1.158;1.328] | 1.298 | [1.218;1.384] |
| Hypertension | 1.262 | [1.168;1.363] | 1.448 | [1.365;1.537] |
| Liver diseases | 1.172 | [1.084;1.267] | 1.185 | [1.094;1.283] |
| Immunodeficiencies | 1.395 | [1.181;1.647] | 1.242 | [1.021;1.511] |
| Malignant growth | 1.307 | [1.219;1.402] | 1.405 | [1.313;1.505] |
| In-situ neoplasm | 0.860 | [0.756;0.977] | 0.837 | [0.710;0.986] |
| Benign neoplasm | 0.858 | [0.807;0.912] | 0.903 | [0.838;0.973] |
| Neoplasm of uncertain behavior | 1.262 | [1.146;1.391] | 1.444 | [1.285;1.622] |
| Depression | 1.022 | [0.956;1.092] | 1.194 | [1.119;1.274] |
| *Comedications* |  |  |  |  |
| ACE | 1.043 | [0.967;1.125] | 0.998 | [0.940;1.061] |
| ARB+RI | 0.935 | [0.866;1.010] | 0.934 | [0.879;0.992] |
| Immunosuppressants | 1.480 | [1.262;1.736] | 1.401 | [1.215;1.615] |
| SSRI | 1.044 | [0.938;1.161] | 0.993 | [0.908;1.085] |
| Tricyclic antidepressants | 1.127 | [1.000;1.271] | 1.086 | [0.985;1.197] |
| Other antidepressants | 1.130 | [1.023;1.248] | 1.116 | [1.027;1.211] |
| Vitamin K antagonists | 1.189 | [1.035;1.367] | 1.071 | [0.949;1.207] |
| Platelet aggregation inhibitors | 1.015 | [0.930;1.108] | 0.991 | [0.920;1.068] |
| Direct factor Xa inhibitors | 1.275 | [1.168;1.392] | 1.043 | [0.971;1.121] |
| Antibiotics | 1.018 | [0.966;1.074] | 1.048 | [1.003;1.095] |
| Metformin | 1.221 | [1.082;1.377] | 1.243 | [1.128;1.369] |
| *Grade of care* |  |  |  |  |
| Care grade 1 | 2.014 | [1.628;2.491] | 2.184 | [1.831;2.606] |
| Care grade 2 | 1.528 | [1.360;1.716] | 2.002 | [1.820;2.202] |
| Care grade 3 | 1.508 | [1.351;1.683] | 2.036 | [1.852;2.238] |
| Care grade 4 | 1.148 | [1.015;1.298] | 1.759 | [1.587;1.950] |
| Care grade 5 | 1.264 | [1.083;1.475] | 1.803 | [1.566;2.076] |

Note: Study period 1: H1/2020, study period 2: H2/2020.

Table S6b: Results of the multivariate logistic regression. Study cohort 2. Study periods 3 and 4.

|  | Study period 3 (n = 38 834) | | Study period 4 (n = 27 762) | |
| --- | --- | --- | --- | --- |
|  | OR | 95%CI | OR | 95%CI |
| *Comorbidities* |  |  |  |  |
| Diabetes | 1.146 | [1.070;1.227] | 1.179 | [1.076;1.291] |
| COPD | 1.172 | [1.077;1.275] | 1.049 | [0.938;1.174] |
| Asthma | 1.027 | [0.958;1.100] | 1.022 | [0.937;1.116] |
| Other chronic respiratory diseases | 0.977 | [0.894;1.067] | 1.166 | [1.025;1.326] |
| Ischemic heart disease | 1.127 | [1.060;1.198] | 1.168 | [1.079;1.264] |
| Hypertension | 1.189 | [1.115;1.269] | 1.241 | [1.142;1.349] |
| Liver diseases | 1.176 | [1.100;1.257] | 1.133 | [1.038;1.235] |
| Immunodeficiencies | 1.320 | [1.129;1.544] | 1.371 | [1.121;1.677] |
| Malignant growth | 1.192 | [1.119;1.270] | 1.229 | [1.132;1.335] |
| In-situ neoplasm | 0.951 | [0.844;1.072] | 0.742 | [0.634;0.869] |
| Benign neoplasm | 0.911 | [0.864;0.960] | 0.931 | [0.871;0.994] |
| Neoplasm of uncertain behavior | 1.281 | [1.173;1.398] | 1.228 | [1.101;1.370] |
| Depression | 1.055 | [0.996;1.118] | 1.320 | [1.229;1.417] |
| *Comedications* |  |  |  |  |
| ACE | 1.093 | [1.022;1.169] | 1.019 | [0.931;1.115] |
| ARB+RI | 1.012 | [0.946;1.083] | 1.046 | [0.958;1.142] |
| Immunosuppressants | 1.320 | [1.140;1.527] | 1.660 | [1.390;1.984] |
| SSRI | 1.208 | [1.096;1.330] | 1.217 | [1.074;1.379] |
| Tricyclic antidepressants | 1.282 | [1.154;1.424] | 1.163 | [1.015;1.333] |
| Other antidepressants | 1.252 | [1.142;1.372] | 1.357 | [1.204;1.529] |
| Vitamin K antagonists | 1.149 | [1.003;1.317] | 1.178 | [0.987;1.406] |
| Platelet aggregation inhibitors | 1.128 | [1.041;1.222] | 1.105 | [0.991;1.232] |
| Direct factor Xa inhibitors | 1.174 | [1.084;1.273] | 1.356 | [1.222;1.505] |
| Antibiotics | 1.173 | [1.118;1.231] | 1.492 | [1.401;1.590] |
| Metformin | 1.245 | [1.123;1.381] | 1.044 | [0.912;1.195] |
| *Grade of care* |  |  |  |  |
| Care grade 1 | 2.304 | [1.901;2.792] | 2.279 | [1.805;2.878] |
| Care grade 2 | 2.547 | [2.283;2.841] | 3.243 | [2.817;3.734] |
| Care grade 3 | 2.804 | [2.515;3.127] | 3.827 | [3.293;4.448] |
| Care grade 4 | 2.710 | [2.385;3.080] | 3.811 | [3.185;4.559] |
| Care grade 5 | 2.707 | [2.244;3.265] | 4.416 | [3.350;5.821] |

Note: Study period 3: H1/2021, study period 4: H2/2021.

Table S6c: Results of the multivariate logistic regression. Study cohort 2. Study periods 5 and 6.

|  | Study period 5 (n = 69 892) | | Study period 6 (n = 76 272) | |
| --- | --- | --- | --- | --- |
|  | OR | 95%CI | OR | 95%CI |
| *Comorbidities* |  |  |  |  |
| Diabetes | 1.221 | [1.150;1.295] | 1.156 | [1.100;1.216] |
| COPD | 1.281 | [1.192;1.376] | 1.286 | [1.208;1.370] |
| Asthma | 1.011 | [0.954;1.071] | 0.922 | [0.874;0.974] |
| Other chronic respiratory diseases | 1.149 | [1.062;1.244] | 1.205 | [1.126;1.290] |
| Ischemic heart disease | 1.224 | [1.163;1.287] | 1.127 | [1.080;1.176] |
| Hypertension | 1.312 | [1.242;1.387] | 1.295 | [1.232;1.362] |
| Liver diseases | 1.250 | [1.179;1.324] | 1.140 | [1.084;1.199] |
| Immunodeficiencies | 1.598 | [1.409;1.813] | 1.576 | [1.403;1.769] |
| Malignant growth | 1.440 | [1.368;1.516] | 1.269 | [1.215;1.326] |
| In-situ neoplasm | 0.798 | [0.725;0.879] | 0.788 | [0.729;0.851] |
| Benign neoplasm | 0.860 | [0.824;0.897] | 0.841 | [0.808;0.874] |
| Neoplasm of uncertain behavior | 1.342 | [1.250;1.440] | 1.347 | [1.266;1.433] |
| Depression | 1.428 | [1.363;1.497] | 1.325 | [1.268;1.384] |
| *Comedications* |  |  |  |  |
| ACE | 1.024 | [0.967;1.084] | 1.040 | [0.989;1.093] |
| ARB+RI | 0.877 | [0.828;0.928] | 0.949 | [0.903;0.996] |
| Immunosuppressants | 2.180 | [1.937;2.452] | 1.765 | [1.588;1.962] |
| SSRI | 1.379 | [1.274;1.492] | 1.255 | [1.165;1.352] |
| Tricyclic antidepressants | 1.300 | [1.189;1.421] | 1.260 | [1.164;1.363] |
| Other antidepressants | 1.328 | [1.232;1.432] | 1.371 | [1.28;1.4690] |
| Vitamin K antagonists | 1.234 | [1.093;1.394] | 1.460 | [1.319;1.616] |
| Platelet aggregation inhibitors | 1.118 | [1.045;1.196] | 1.209 | [1.142;1.281] |
| Direct factor Xa inhibitors | 1.329 | [1.247;1.416] | 1.325 | [1.258;1.396] |
| Antibiotics | 1.344 | [1.293;1.397] | 1.242 | [1.197;1.288] |
| Metformin | 1.053 | [0.963;1.151] | 1.118 | [1.036;1.207] |
| *Grade of care* |  |  |  |  |
| Care grade 1 | 2.584 | [2.243;2.977] | 2.472 | [2.217;2.758] |
| Care grade 2 | 3.338 | [3.062;3.638] | 3.430 | [3.197;3.680] |
| Care grade 3 | 3.683 | [3.379;4.013] | 4.132 | [3.840;4.446] |
| Care grade 4 | 3.321 | [3.003;3.673] | 4.132 | [3.770;4.530] |
| Care grade 5 | 3.783 | [3.274;4.371] | 4.129 | [3.590;4.748] |

Note: Study period 5: H1/2022, study period 6: H2/2022.

Figure S1: Association between grade of care and risk of hospitalisation with COVID-19. Study cohort 1.

Notes: Estimates are based on a multivariate conditional logistic regression adjusted for comorbidities, comedications, and grade of care. For further details, refer to the methods section in the paper.

Figure S2: Association between grade of care and risk of hospitalisation with COVID-19. Study cohort 2.

Notes: Estimates are based on a multivariate conditional logistic regression adjusted for comorbidities, comedications, and grade of care. For further details, refer to the methods section in the paper.
